# Customizing 2.5D Out‐of‐Plane Architectures for Robust Plasmonic Bound‐States‐in‐the‐Continuum Metasurfaces

**DOI:** 10.1002/advs.202206236

**Published:** 2023-01-03

**Authors:** Zichen Wang, Jiacheng Sun, Jiye Li, Lang Wang, Zishun Li, Xiaorui Zheng, Liaoyong Wen

**Affiliations:** ^1^ Zhejiang University Hangzhou Zhejiang 310027 P. R. China; ^2^ Research Center for Industries of the Future (RCIF) School of Engineering Westlake University Hangzhou Zhejiang 310030 P. R. China; ^3^ Key Laboratory of 3D Micro/Nano Fabrication and Characterization of Zhejiang Province School of Engineering Westlake University Hangzhou Zhejiang 310030 P. R. China

**Keywords:** 2.5D out‐of‐plane architecture, binary‐pore anodic aluminum oxide template, biosensing, bound states in the continuum, deep neural network, plasmonic metasurface

## Abstract

Bound states in the continuum (BICs) have a superior ability to confine electromagnetic waves and enhance light–matter interactions. However, the quality‐factor of quasi‐BIC is extremely sensitive to structural perturbations, thus the BIC metasurfaces usually require a very‐high precision nanofabrication technique that greatly restricts their practical applications. Here, distinctive 2.5D out‐of‐plane architectures based plasmonic symmetry protected (SP)‐BIC metasurfaces are proposed, which could deliver robust quality factors even with large structural perturbations. The high‐throughput fabrication of such SP‐BIC metasurfaces is realized by using the binary‐pore anodic aluminum oxide template technique. Moreover, the deep neural network (DNN) is adapted to conduct multiparameter fittings, where the 2.5D hetero‐out‐of‐plane architectures with robust high quality‐factors and figures of merit are rapidly predicted and fabricated. Finally, owning to its large second‐order surface sensitivity, the desired 2.5D hetero‐out‐of‐plane architecture demonstrates a detection limit of endotoxin as low as 0.01 EU mL^‐1^, showing a good perspective of biosensors and others.

## Graphical abstract

A series of 2.5D out‐of‐plane architectures are proposed by using a combination of binary‐pore anodic aluminum oxide template technique and deep neural network to efficiently realize plasmonic symmetry protected bound states in the continuum (BIC) metasurfaces with robust high quality‐factors and figures of merits that show a good perspective of biosensors and others.

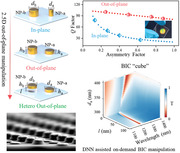

## 1 Introduction

Bound states in the continuum (BICs) are localized states with infinite lifetimes in the presence of a continuous spectrum with matched energy and momentum.^[^ ^1^, ^2^ ^]^ Though BICs were initially proposed in quantum mechanics, due to a universal characteristic of the Schrödinger equation, BICs have been shown to exist in all domains of wave mechanics and are actively being investigated.^[^ ^3^, ^4^, ^5^, ^6^, ^7^, ^8^, ^9^, ^10^, ^11^ ^]^ Due to the lower material loss, *Q‐*factors in dielectric metasurface are usually higher than those in plasmonic,^[^ ^12^ ^]^ but the plasmonic structures also maintain various unique properties, like large electric field enhancement, high Purcell factor, etc.^[^ ^13^, ^14^, ^15^, ^16^, ^17^ ^]^ Therefore, both of dielectric and plasmonic metasurfaces are important research branches in the field of optical‐BICs. And now, many types of those BIC metasurfaces have been proposed for numerous breakthrough applications ranging from biosensing^[^ ^18^, ^19^, ^20^, ^21^, ^22^ ^]^ to higher harmonic generation^[^ ^23^, ^24^, ^25^ ^]^ and lasing.^[^ ^26^, ^27^, ^28^ ^]^ In particular, symmetry protected (SP)‐BIC engineered via symmetry mismatch, has aroused preponderant interest due to its easy predictability and manipulability.^[^ ^29^ ^]^

The perfect SP‐BIC can be regarded as a bound eigenmode with an infinite quality‐factor (*Q‐*factor) but cannot be observed in the far‐field condition, which means that above the photonic light line, the SP‐BIC is a resonance with disappeared linewidth. When the ideal BIC is disturbed, it provides a channel for energy leaking to the free space with a finite *Q‐*factor that is defined as quasi‐BIC (q‐BIC). So far, abundant classic 2D in‐plane (2D IP) architecture designs, including tilted ellipses,^[^ ^20^, ^30^ ^]^ asymmetric rods,^[^ ^31^ ^]^ split rings,^[^ ^32^, ^33^ ^]^ and so on,^[^ ^34^, ^35^, ^36^, ^37^, ^38^ ^]^ have been experimentally realized with advanced nanofabrication techniques. However, their q‐BIC resonances are extremely sensitive to the in‐plane structural perturbations that their *Q‐*factors decay rapidly with the increase of the asymmetry factor (AF, or *α*) of IP architectures.^[^ ^39^ ^]^ Moreover, the q‐BIC resonance also shows very low tolerance to the defects, and few nanometers imperfection will dramatically reduce the *Q‐*factors and weaken the resonances.^[^ ^40^ ^]^ Thus, a satisfactory q‐BIC resonance generally can only be selected within an extremely narrow structural manipulating window,^[^ ^40^, ^41^, ^42^, ^43^ ^]^ which inevitably requires the enormous costs and greatly limits the application prospect of q‐BIC in diverse photonics devices. So, the following question is that: is there an appropriate strategy to realize a structural‐asymmetry BIC metasurface with a robust *Q*‐factor?

Considering the limitations of the 2D IP architectures, possible solutions are sought in higher dimensions of the BIC metasurfaces.^[^ ^29^, ^44^, ^45^ ^]^ Such as, by microprinting a 3D photonic crystal structure (3D PhC) using two‐photon polymerization, a line of BICs with a “symmetry bandgap” instead of a point was obtained, which can provide a wide range of high‐*Q* states, making the BIC PhCs a more robust high‐*Q* device.^[^ ^29^ ^]^ Nonetheless, fabrication of such complex 3D PhC is very slow which restricts its scalable application.^[^ ^46^ ^]^ On the other hand, 2.5D out‐of‐plane (OP) architecture, such as a pair of resonators with different thicknesses, is a relatively simple design and could be cost‐effectively achieved for pursuing robust SP‐BIC metasurface, but very limited attention has been paid to this configuration yet.^[^ ^47^, ^48^ ^]^

In this work, we demonstrated plasmonic SP‐BIC metasurfaces with robust *Q*‐factors via manipulating a series of novel 2.5D OP architectures based on the binary‐pore anodic aluminum oxide (BP‐AAO) template technique. The finite‐difference time‐domain (FDTD) simulation and experimental results confirmed that as the AF (*α*) increases, the sole 2.5D OP architectures with different thicknesses of Au nanoparticles (NPs) arrays show a much narrower and more stable linewidth of q‐BIC resonances along with a quasi‐linear decay rate of *Q*‐factor, in contrast to that a dramatical decay rate of *Q*‐factor based on the conventional sole 2D IP architectures. The reason can be attributed to the polarizability difference between the two plasmonic metal NPs in the OP and IP architectures. Moreover, the deep neural network (DNN) was employed to assist the multiparameter fittings and fast prediction of SP‐BIC metasurfaces. The desired hetero‐out‐of‐plane (H‐OP, Au‐SiO_2_) architecture was proposed, which possesses the advantages of a robust high *Q‐*factor and figure of merit (FoM) that is very suitable for biosensing. The analysis of bulk and surface sensing performance proved that the H‐OP architecture possesses a large second‐order surface sensitivity. Thereout, its detection limit to the endotoxin can be as low as 0.01 EU mL^‐1^ which is superior to the most commercialized kits. Overall, the proposed 2.5D architectures could provide inspiration for fabricating new types of practical SP‐BIC metasurfaces on a large scale for optical and optoelectronic devices.

## 2 Results and Discussion

### 2.1 Design of 2.5D OP Architectures

**Figure 1:**
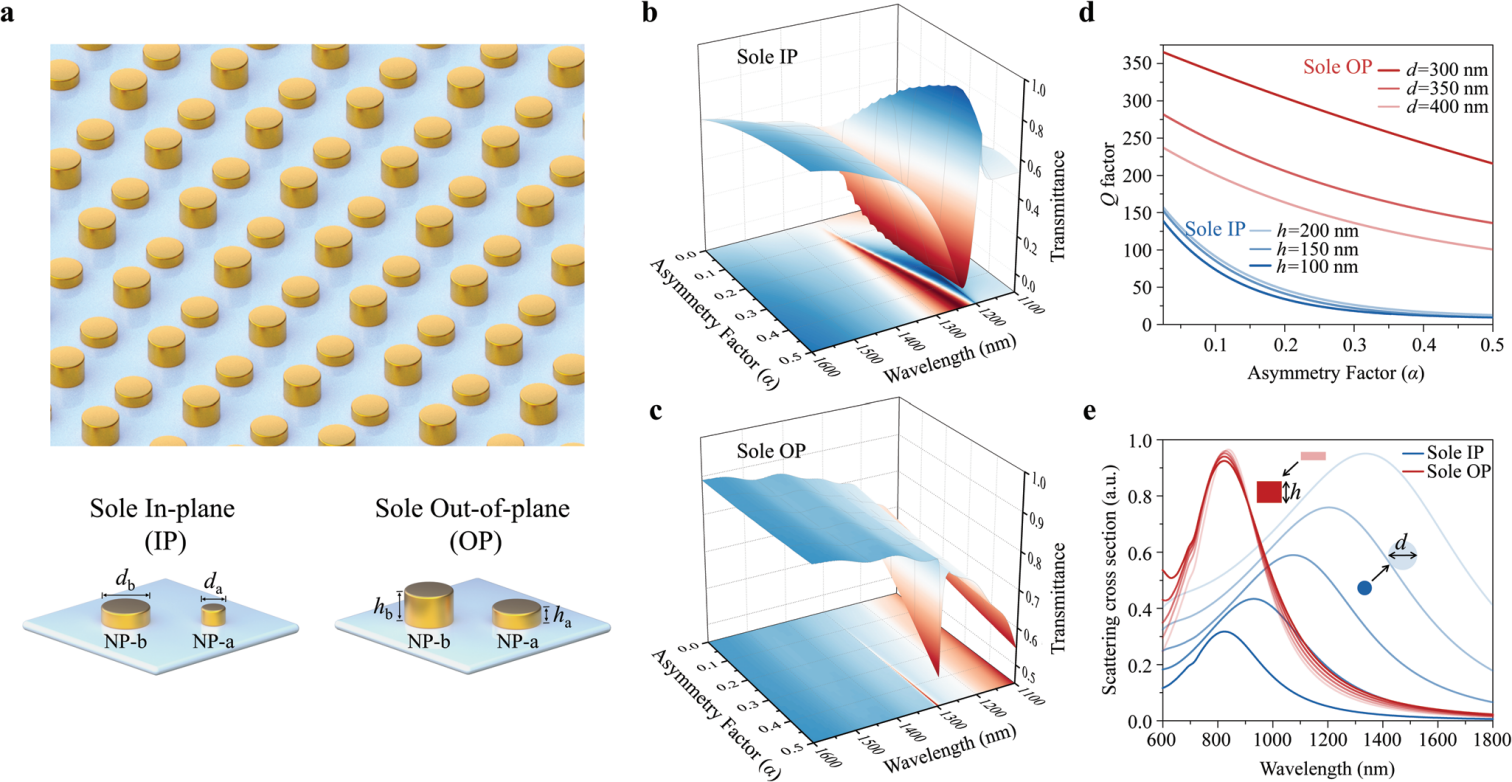
Sole IP and OP architectures based plasmonic SP‐BIC metasurfaces. a) Schematic of metasurfaces with sole IP symmetry‐broken (Δ*d*) and sole OP symmetry‐broken (Δ*h*). b)Calculated transmittance spectra of sole IP architectures with *h* = 100 nm for all NPs, where the *d*_b_ of NPs‐b is fixed to 300 nm while the *d*_a_ of NPs‐a is reduced from 300 nm to 150 nm. c)Calculated transmittance spectra of sole OP architectures with *d* = 300 nm for all NPs, where the *h*_b_ of NPs‐b is 200 nm while *h*_a_ of NPs‐a is collapsed from 200 to 100 nm. d) Calculated *Q*‐factors as a function of *α* for sole IP and OP architectures. The blue lines: sole IP architectures with different *h* from 100 nm to 200 nm fitted by exponential curves: *Q =* 164.63 exp(‐*α*/0.11) + 7.58 (*h* = 100 nm); *Q =* 178.84 exp(‐*α*/0.12) + 6.44 (*h* = 150 nm); *Q =* 180 exp(‐*α*/0.13) + 8.96 (*h* = 200 nm). The red lines: sole OP architectures with different *d* from 300 nm to 400 nm fitted by exponential curves: *Q =* 524.56 exp(‐*α*/1.39) ‐ 149.94 (*d* = 300 nm); *Q =* 216.36 exp(‐*α*/0.37) + 79.41 (*d* = 350 nm); *Q =* 193.86 exp(‐*α*/0.33) + 57.23 (*d* = 400 nm). e) Scattering cross sections (*σ* ^sca^) of single Au NP on SiO_2_ substrate. Red lines: *d* is fixed to 200 nm and *h* is tailored from 60 nm to 140 nm. Blue lines: *h* is fixed to 100 nm and *d* is tailored from 200 to 400 nm.

The plasmonic SP‐BIC metasurfaces with 2.5D OP and 2D IP architectures are depicted in **Figure** **1**a. It is a periodic matrix with repetition of a supercell containing two Au NPs over a square lattice (period *T* = 800 nm). These two Au NPs are named as the NPs‐a and NPs‐b, and every NPs‐b (or a) is situated at the fourfold junction site of the NPs‐a (or b). At the SP‐BIC point, NPs‐a (diameter *d*_a_ & height *h*_a_) and NPs‐b (diameter *d*_b_ & height *h*_b_) have completely equivalent geometry. Moreover, for the sole IP architectures, we fix the Δ*h* (*h*_b_ ‐ *h*_a_) at zero and only adjust the Δ*d* (*d*_b_ ‐ *d*_a_); while for the sole OP architectures, we fix the Δ*d* at zero and only adjust the Δ*h*. In other words, the manipulation of the IP architectures is limited to the *X*‐*Y* plane, while it extends to *Z‐*direction for the OP architectures, as shown at the bottom of Figure 1a. For convenience, we use AF (*α*) to describe the variation of these geometric parameters. For all metasurfaces, the IP AF is defined as Δ*d*/*d*_b_ and the OP AF is defined as Δ*h*/*h*_b_. Here, the OP (or IP) AF of the IP (or OP) architectures is equal to zero, so we can consider the IP (or OP) AF as the ultimate AF of the IP (or OP) architectures.

By using full‐wave numerical analysis, the transmission spectra of metasurfaces with sole IP and sole OP architectures were calculated (Figure 1b,c). Two types of q‐BIC resonances manipulations behave obviously differ from each other. For the sole IP manipulation, it can be observed that the resonance intensity of q‐BIC excited from IP architecture grows dramatically with the rapid broadening of linewidth and prominent blue‐shift of the resonance peak. However, for the sole OP manipulation, impressively, with the increase of *α*, the q‐BIC excited from sole OP architecture maintains a very sharp peak and stable resonance wavelength even with a large *α*, revealing that a very limited radiative loss is introduced. *Q*‐factor (*Q* = *λ*/FWHM, where FWHM is the full width of half maximum) as the function of *α* is calculated for both sole IP and OP architectures with different *h* and *d*, respectively, and they are fitted with exponential function *Q* = *A*_exp_(‐*α*/*τ*) + *y*_0_ in Figure 1d. The increase of *h* doesn't make big difference for improving the fast decay (*τ* ≈ 0.12) and low value of *Q‐*factor (<160) in sole IP manipulation (the blue lines). On the contrary, the red lines represent the sole OP manipulation which exhibits a much slower *Q* factor degeneration with *τ* reaches to 1.39 and higher *Q* factor values more than 300. Even when the *d* increases from 300 nm to 400 nm, bringing a larger nonradiative loss, the overall *Q*‐factor value of sole OP architecture is still larger than that of the sole IP architecture.

The robust q‐BIC resonance behavior originated from sole OP manipulation can be explained by the moderate polarizability difference between two metal NPs in a 2.5D symmetry‐broken binary plasmonic NPs array.^[^ ^49^ ^]^ Since our metasurfaces can support subradiant lattice resonance whose radiative loss *γ* is proportional to the polarizability difference Δ*p* between NP‐a and NP‐b: *γ* ≈ ‐Im(Δ*p*)^2^. When the Δ*p* reaches 0, *γ* is completely suppressed and the resonance turns into a perfectly dark mode (BIC) with vanished linewidth. When the structural symmetry is broken, the variation of Δ*p* will generate q‐BIC resonance with a finite *Q*‐factor due to the radiative loss. According to the definition of polarizability (see Experimental section),^[^ ^50^ ^]^ when the structural perturbation of supercells is changed parallelly to the applied electric field (or polarization), the Δ*p* of NPs will be remarkably modified for the IP architecture, which will make a sharp degeneration of the *Q*‐factor (*Q* ≈ 1/*γ*); however, if the particle is only manipulated along the wave vector direction (OP), the Δ*p* will be generated much more gently as the function of *α*, which results in a quasi‐linear degeneration of the *Q*‐factor. To further verify our hypothesis, we calculated the scattering cross section (*σ* ^sca^ = 8*πk* ^4^|*p*|^2^/3) of NPs with different *d* and *h* normally irradiated by unpolarized light. As shown in Figure 1e, when *h* of a single NP increases (red lines), the *σ* ^sca^ peak wavelength shifts slightly, and the intensity degenerates subtly; however, when *d* of a single NP increases (blues lines), the *σ* ^sca^ peak shows obvious red‐shift and broadening, and its intensity increases dramatically. These results imply the fact that by changing *h*, the *p* can be manipulated in a more meticulously way even with large parameter perturbations. Therefore, a new type of plasmonic SP‐BIC metasurface based on the 2.5D OP manipulation was proposed, which allows a more robust *Q*‐factor compared to that of the 2D IP manipulation, and such conceive is visualized in the following experimental part.

### 2.2 Experimental Demonstration of 2.5D OP Architectures

**Figure 2:**
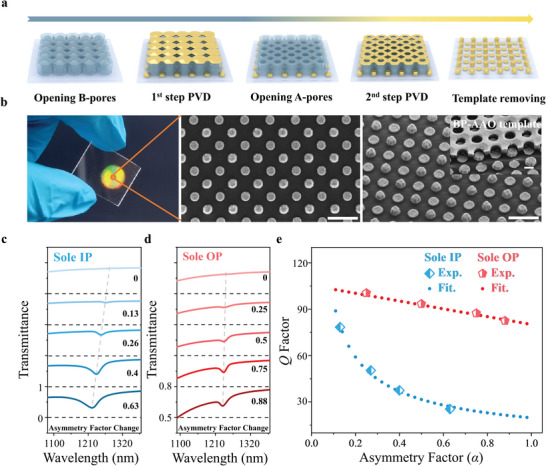
Fabrication and optical characterization of plasmonic SP‐BIC metasurfaces. a) Schematic fabrication process of the sole OP architectures. b) Photograph and SEM images of the representative sole OP architectures (Scale bars: 1 µm). The BP‐AAO template after two‐step PDV processes is shown in the inset SEM image (scale bar: 250 nm). Experimental transmission spectra of c) sole IP architectures with different *α* (for all samples, *h*_a_ = *h*_b_ = 100 nm and *d*_b_ = 300 nm, while Δ*d* are 0, 40 nm, 80 nm, 120 nm and 190 nm with their corresponding *α* of 0, 0.13, 0.27, 0.4 and 0.63, respectively) and d) sole IP architectures (for all samples, *d*_a_ = *d*_b_ = 300 nm and *h*_b_ = 160 nm, while Δ*h* are 0, 40 nm, 80 nm, 120 nm and 140 nm with their corresponding *α* of 0, 0.25, 0.5, 0.75 and 0.88, respectively). e) Related variation trends of *Q*‐factor with the increasing of *α* for sole IP (the blue curve) and OP (the red curve) architectures.

The BP‐AAO template technique was employed to demonstrate the characteristics of plasmonic SP‐BIC in sole IP and OP architectures experimentally.^[^ ^51^ ^]^ Due to the unique structure of two barrier layers, the two subset nanopores of BP‐AAO template can be opened and adjusted individually, allowing the multidimensional regulations (size, height, and material) of the two subset components (named NPs‐a and NPs‐b) with a perfect self‐alignment. The representative fabrication process of the sole OP architecture is shown in **Figure** **2**a and Figure S1 (Supporting Information). It is worth noting that the “OP” is used to describe the metasurface with Δ*h* and homogeneous NPs‐b, while “H‐OP” is used to describe the metasurface with Δ*h* and hybrid NPs‐b (Figure S2, Supporting Information). The photograph of representative sole OP architecture and its corresponding (scanning electron microscopy) SEM images are shown in Figure 2b, where both sets of NPs are periodically arranged in the tetragonal form with an identical size but different heights. For the sole IP architecture, we only changed the Δ*d* between NP‐a and NP‐b and kept their Δ*h* unchanged. Based on this criterion, we prepared various sole IP architectures with *α* of 0, 0.13, 0.27, 0.4, and 0.63, and sole OP architectures with *α* of 0, 0.25, 0.5, 0.75 and 0.88, respectively (Figures S3 and S4, Supporting Information).

The measured transmission spectra are shown in Figure 2c,d, when *α* = 0, the geometric symmetry of NPs‐a and NPs‐b allows destructive interference between the two particles, resulting in a lossless resonance that cannot be observed from the far field, which is an SP‐BIC. While as *α* gradually increases, the perfect bound state starts to generate radiative loss, which is identified as linewidth broadening and intensity enhancement of q‐BIC resonance for both sole IP and OP architectures. The difference is that, for the sole IP architectures, the transmission peaks tend to be fast blue‐shifted by increasing the *α*, and accompanied by a great broadening of the FWHMs and enhancement of the peak intensities. But for sole OP architectures, even with a large height difference (≈120 nm), the q‐BIC maintains a narrow peak and stable resonance wavelength, coincident with the simulation results in Figure 1e. We summarized the *Q*‐factor variation trends of the two types of architectures with the increase of *α* (Figure 2e). For the sole IP architectures, the *Q*‐factor decreases exponentially as the function of *α*, while the *Q*‐factor of sole OP architectures decreases almost linearly with the increase of *α*, showing outstanding robustness. In conclusion, our experimental results indicate that the decay rules between the *Q*‐factor and the *α* of these SP‐BIC metasurfaces are consistent with the prediction of simulations, stating our hypothesis that the sole OP architecture possesses a more robust *Q‐*factor. In addition, even if the volume difference is used to define the AF_(_ *_V_*_)_, similar conclusions can still be drawn (Figure S5, Supporting Information).

### 2.3 Analysis of BIC Metasurface with DNN Algorithm

**Figure 3:**
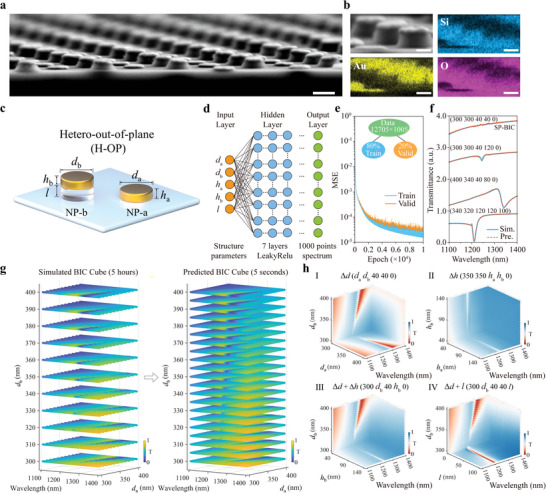
Analysis of plasmonic SP‐BIC metasurface based on DNN algorithm. a) Cross‐sectional SEM image of a representative H‐OP architecture. Scale bar: 300 nm. b) The element mapping of correlative NPs in the H‐OP architecture. All scale bars: 100 nm. c) Schematic of single asymmetric meta‐atom in the H‐OP architecture. d) Deep‐learning model of optical response from 5 input structural parameters. e) The learning curve of DNN. f) Typical DNN model predicted spectra compared with ground truth simulation results. g) Analysis efficiency comparison between traditional full‐wave numerical simulation and trained DNN model. The left column is the BIC spectral cube as the function of *d*_a_ and *d*_b_ from 300 nm to 400 nm in the step of 10 nm, taking 5 h for calculation. The right column is the BIC spectral cube as the function of *d*_a_ and *d*_b_ from 300 to 400 nm in the step of 5 nm, taking only 5 s for calculation. h) BIC spectral cubes as the function of [*d*_a_ *d*_b_], [*h*_a_ *h*_b_], [*d*_b_ *h*_b_], and [*d*_b_ *l*].

Moreover, benefiting from the distinctive BP‐AAO template, we can also produce architectures with multi‐materials in one matrix. Such as, by depositing SiO_2_ in the B‐pores of the BP‐AAO template first, and then followed by Au deposition after opening the A‐pores, two Au particles with the same geometry but at the different spatial positions can also introduce energy leakage and radiative loss into the BIC system, leading to the generation of quasi‐BIC resonance (**Figure** **3**a,b and Figures S6 and S7, Supporting Information). Such an architecture endows 5‐dimensions to manipulate the BIC behavior, which is illustrated in Figure 3c: the diameter of particles a and b (*d*_a_, *d*_b_), the height of particles a and b (*h*_a_, *h*_b_), and the thickness of SiO_2_ (*l*). Considering the complexity of analyzing such 2.5D H‐OP architecture, traditional full‐wave numerical methods like FDTD would be too time‐consuming and burdensome. Alternatively, DNN‐based nanophotonic structure design is a promising approach nowadays, which can offer a more efficient tool to investigate the multi‐dimensional BIC behavior.^[^ ^52^ ^]^ By applying a seven‐layers fully connected neural network (FCNN) with the parameters of 5D structure as input and a high resolution 1000 points transmittance spectrum from 1100 to 1400 nm as output, we ran 12 705 simulations to construct the dataset, and only 80% of them were required for a well‐converged model training (the training data can even be much smaller, see Figure S8, Supporting Information) as illustrated in Figure 3d,e. More detailed information about the DNN algorithm can be found in the Experimental section.

The trained DNN model shows remarkable accuracy in the prediction. One can observe in Figure 3f that 4 typical predicted spectra lines with distinct structure parameters match very well with the FDTD simulation results. This proves that our model is reliable for predicting optical response either at different wavelengths or with various shapes. Based on this well‐established model, we employed it for big data analysis to explore the more fascinating phenomena in higher dimensional structures. By simultaneously sweeping *d*_a_ and *d*_b_ in Figure 3g, we obtained a series of spectra mappings and each of them demonstrated the variation of BIC with the change of structural parameters. Here, we named such a 3D set of BIC mappings as “BIC cube.” With a step of 10 nm, using FDTD simulation to calculate such a BIC cube needs at least 5 hours (CPU i9‐12900k, Memory 64 GB, FDTD mesh 5 nm), and the data is still sparse as shown in the left column of Figure 3g, while it only takes 5 s for the DNN model with a finer sweeping step of 5 nm to provide a much more elaborated BIC cube in the right column of Figure 3g. With the assistance of DNN, we can observe the existence of an SP‐BIC line in the structure parameter space, thus a wide range of high‐*Q* q‐BIC resonances can be obtained around this SP‐BIC line, which endows more freedom to manipulating. Furthermore, we calculated the BIC spectral cube as the function of [*d*_a_ *d*_b_], [*h*_a_ *h*_b_], [*d*_a_ *h*_b_], and [*d*_b_ *l*] in Figure 3h. These results reveal the diversified BIC manipulation brought by multidimensional parameters. For example, though the Δ*h* can bring a high *Q*‐factor, the intensity of resonance is remarkably weak as shown in the second cube in Figure 3h. Nevertheless, once combining the Δ*d* and Δ*h*, as shown in the third cube in Figure 3h, both advantages of the high intensity in sole IP architecture and the high *Q*‐factor in sole OP architecture can be integrated for an optimized strong resonance. And in the fourth cube of Figure 3h, with the addition of *l*, the H‐OP architecture can provide a strong, narrow, and robust q‐BIC peak simultaneously. In this way, we no longer need to use metasurface with a large sole OP AF for producing a robust high‐*Q* resonance, which could save a large amount of Au usage. Besides, we also noticed that the q‐BIC resonance induced by H‐OP architecture is very sensitive to the *d*_a_ but is insensitive to the *d*_b_, which means that with a fixed *d*_a_, the peak of the H‐OP architecture would be very stable to the variation of *d*_b_ (Figure S9, Supporting Information). These multi‐parameters predictions are very helpful to guide and optimize the experimental results.

### 2.4 Realization of Quasi‐BIC Resonances Based on 2.5D H‐OP Architectures

**Figure 4:**
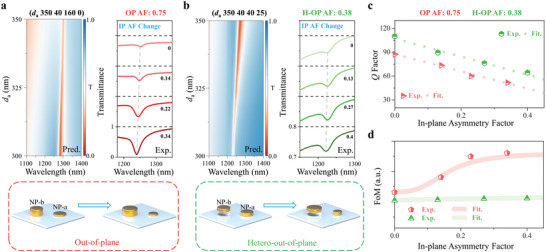
Realization of desirable quasi‐BIC resonances based on H‐OP architectures. a) Left is the spectral contour of input structural parameter (*d*_a_ 350 40 160 0), where *d*_a_ is adjusted from 300 to 350 nm. Right is the experimental spectra of the SP‐BIC metasurface with a fixed OP AF of 0.75 and changing the IP AF from 0 to 0.34. b) Left is the spectral contour of input structural parameter (*d*_a_ 350 40 40 25) where *d*_a_ is adjusted from 300 to 350 nm. Right is the experimental spectra of the SP‐BIC metasurface with a fixed H‐OP AF of 0.38 and changing the IP AF from 0 to 0.4. c,d) The experimental *Q*‐factor and the normalized FoM of the SP‐BIC metasurfaces as the function of IP AF with a fixed OP AF of 0.75 and H‐OP AF of 0.38, respectively.

As it is recognized that there is a trade‐off between the *Q*‐factor and the intensity of quasi‐BIC resonance, a high *Q*‐factor with a low intensity has measuring difficulty with low resolution and poor signal‐to‐noise system, which will restrain its performance in applications like biomolecular sensing or imaging.^[^ ^53^ ^]^ DNN‐assistant multi‐dimensional manipulation endows us with an efficient tool to pursue q‐BIC resonance with a strong FoM (FoM = *Q* × *I*, *I* is the resonance intensity extracted from the resonance peak and dip).^[^ ^22^ ^]^ From the predicted spectral mapping shown in **Figure** **4**a (left), the q‐BIC resonance could obtain a fast increase of intensity by introducing Δ*d* with a variation of *d*_a_ while maintaining a sharp linewidth by fixing Δ*h* to a suitable value. The same phenomena are also observed in experimental results (right of Figure 4a, Figures S10 and S11, Supporting Information), with a fixed OP AF of 0.75, the increase of IP AF leads to a deeper q‐BIC peak and little linewidth widening that could overcome the weakness of sole IP architecture. Moreover, by introducing the Δ*d* with a variation of *d_a_* into the H‐OP architecture, the q‐BIC resonance becomes even sharper in the predicted mapping and the corresponding experimental spectra exhibit robust line shapes with a fixed H‐OP AF of 0.38 (Figure 4b). Therefore, the *Q*‐factors as the function of IP AF with a fixed OP AF of 0.75 and H‐OP AF of 0.38 are calculated in Figure 4c, which both exhibit a similar quasi‐linear degeneration. By considering both the *Q*‐factor and peak intensity, the corresponding FoM are calculated in Figure 4d, where the FoM of OP architecture has a persistently positive correlation with the IP AF, while the FoM of H‐OP keeps very robust. It reveals that the q‐BIC resonance with a robust high *Q*‐factor and FoM can be obtained by manipulating both in‐plane and (hetero) out‐of‐plane architectures simultaneously.

### 2.5 Customized 2.5D H‐OP Architectures for Biosensing

**Figure 5:**
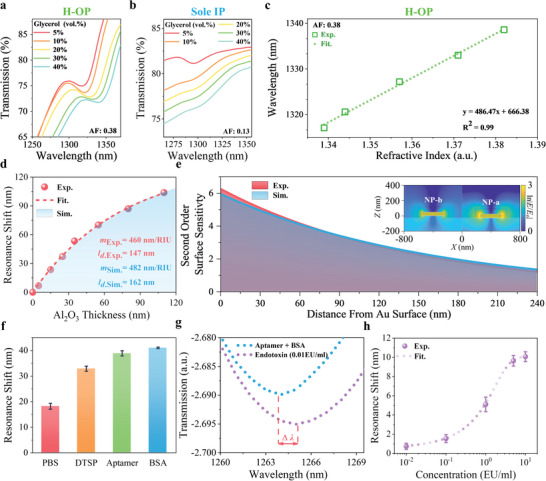
H‐OP architecture based biosensing. Peak shifts of q‐BIC resonance for the a) H‐OP architecture (*α* = 0.38) and b) IP architecture (*α* = 0.13) in glycerol dilutions (5‐40% vol%). c) Experimental q‐BIC resonance wavelength shifts as a function of RI variation. Dotted lines indicate the linear response. d) Experimental and simulated peak position shifts as a function of the Al_2_O_3_ thickness. The red dashed line is fitted from experimental data and the blue region is fitted simulation result according to Equation (3). e) Calculated second‐order surface sensitivity of H‐OP architecture as a function of the distance from H‐OP architecture, where the red and blue areas are experimental and simulated results, respectively. Insets are the electric field distribution of the two Au NPs surfaces at quasi‐BIC resonance illuminated by a plane wave propagating along the +*z* direction. f) q‐BIC resonance shifts of the H‐OP architecture after the sequential modification steps: PBS, DTSP, aptamer, and BSA. g) q‐BIC resonance shift of the H‐OP architecture after the adsorption of endotoxin with a concentration of 0.01 EU mL^‐1^ in PBS buffer solution. h) q‐BIC resonance shifts in response to different endotoxin concentrations (0.01, 0.1, 1, 5 and 10 EU mL^‐1^, corresponding average shifts are 0.7, 1.5, 5.1, 9.6, and 10.1 nm, respectively).

BIC metasurfaces‐based biosensors that utilize the resonance peak shift caused by the binding of specific biomolecules are considered promising candidates for detecting biomolecules with multiplexing and quantification.^[^ ^18^, ^19^, ^20^, ^21^, ^22^ ^]^ To demonstrate the potential of H‐OP architecture as biosensors, we integrated the Au‐SiO_2_ hybrid metasurface (H‐OP AF = 0.38, *Q*‐factor ≈110.1) with a polymethyl methacrylate (PMMA) microfluidic chip (Figure S12, Supporting Information). First, we measured the transmission spectra of the H‐OP architecture in different concentrations of glycerol to calculate the bulk sensitivity. The experimental results are shown in **Figure** **5**a, the concentration of glycerol has varied from 5 to 40 vol%, corresponding to the transmission peak position shifts from 1317.1 to 1338.6 nm. A linear fitting of the transmission peak shifts for RI variations demonstrates the value of bulk sensitivity as about 486 nm/RIU based on Δ*λ* = 21.5 nm and Δ*n* = 0.043 RIU (Figure 5c). In addition, we also investigated the sensing performance of sole IP (IP AF = 0.13) and OP (OP AF = 0.75) architectures, and the experimental results are shown in Figure 5b and Figure S13 (Supporting Information). For the IP sample, we chose this sample near the BIC due to its relatively high *Q*‐factor, but it is very easily disturbed by environmental perturbations due to the small AF (Figure 1b). Its q‐BIC resonance becomes broad and finally almost disappeared as glycerol concentration increases; hence the peak shifts cannot be identified accurately, indicating that the IP architecture with *Q‐*factor (≈78.4) is not robust for the RI sensor. For the OP sample, due to its robust *Q‐*factor (≈87.4) and resonance intensity, its experimental bulk sensitivity is about 471 nm/RIU based on Δ*λ* = 21 nm and Δ*n* = 0.043 RIU. It is worth mentioning that the H‐OP architecture can otherwise significantly reduce the use of precious metals than that compared with the OP architecture, representing a more practical application prospect. Thus, we apply the H‐OP architecture for the further biosensing performance investigation.

Moreover, since the bulk sensitivity cannot fully evaluate the biomolecular sensing performance of nanostructures,^[^ ^54^, ^55^, ^56^, ^57^, ^58^, ^59^, ^60^ ^]^ we further characterized the surface sensitivity of the H‐OP architecture. Specifically, we employed the atomic layer deposition (ALD) to deposit Al_2_O_3_ dielectric layers with different thicknesses and well‐defined refractive indices. It can be seen that the q‐BIC peak tends to be gradually red‐shifted with the increase of Al_2_O_3_ thickness (Figure S14, Supporting Information). According to the curve (red dotted line) fitted from the experimental data, we noticed that the spectral shifts saturated gradually per 30 nm of Al_2_O_3_ depositions, and the similar tendency can also be found in the blue curve fitted by the FDTD simulation which matches very well to the experimental result (Figure 5d), implying that the surface sensitivity of the metasurface is dropping with distance away from the metal surface. This is due to the fact that as the distance from the Au surface increases, the electric field intensity will decay inevitably. Additionally, we calculated the sensitivity factor *m* and decay length *l*_d_ of the H‐OP architecture by using the theoretical equation, the specific calculation process is shown in the Methods. The results obtained from the experiment and the simulation are basically consistent (Figure 5d), *m*_Exp._ and *m*_Sim._ are 460 and 482 nm/RIU, while *l*_dExp._ and *l*_dSim._ are 147 and 162 nm, respectively. It is worth noting that when the thickness of adsorbate layer tends to be infinite, the value of *m* should be derived as the value of the bulk sensitivity.^[^ ^61^ ^]^ By comparing the sensitivity factor *m* obtained from the ALD experiment with the bulk sensitivity obtained from the glycerol experiment, the two values are similar to each other, which are 460 nm/RIU and 486 nm/RIU, respectively. These results confirm that the *m* factor is the bulk sensitivity, verifying the reliability of our experimental results.

In order to reveal the relationship between the sensitivity and the thickness of the Al_2_O_3_ dielectric layers more deeply, we studied the second‐order surface sensitivity of H‐OP architecture. The experimental and simulated results are shown in Figure 5e. The performance of the biosensor can be simply evaluated by the area of the region enclosed by the distance range and the second order sensitivity curve. Generally, the sensitivity decays away from the surface by the factor of exp(−2*t*/*l*_d_), for the detailed calculation process, see Methods. The analogous exponential decay trend can also be seen in the electric field distribution of the two Au NPs surfaces in the inset of Figure 5e. In the FDTD simulation model, Au NP‐a and NP‐b are both surrounded by Al_2_O_3_ dielectric layers, strongest electric field strengths are observed on the flank of the Au NPs and decay exponentially with the distance increasing. In addition, we also noticed that according to the theoretical calculation results, the value of second‐order surface sensitivity reaches about 6 when the adsorbate thickness *t* = 0, and even when the *t* is greater than 240 nm, it still maintains a value larger than 1. Such a slow sensitivity decay rate benefits from the larger decay length (*l*_d_ ≈ 150 nm) of the H‐OP architecture, which may be the strong light–matter interaction induced by q‐BIC resonance.

Considering the value of the second‐order surface sensitivity of the H‐OP architecture only decrease about 4% when the adsorbate thickness is about 5 nm, implying that the H‐OP architecture may have the potential to efficiently detect small biological molecules. Therefore, the H‐OP architecture was used to identify endotoxin as an example (Experimental section and Figure S16, Supporting Information). It can be seen that after sequential modification steps, the q‐BIC peak undergoes obviously red‐shifts (Figure S17, Supporting Information), indicating that the modification steps have been carried out successfully. We calculated the resonance shifts by using three groups of experiments, and the associated mean values and errors are shown in Figure 5f and Table S1 (Supporting Information). The average resonance shifts of the H‐OP architecture in PBS solution and modified with DTSP, aptamer, and BSA are 18.2, 32.9, 38.9, and 40.9 nm, respectively. After these modification steps, the average resonance shifts of target analytes and errors at different concentrations are shown in Figure 5h and Table S2 (Supporting Information), the stipple curve was fitted using the logistic5 model with a correlation coefficient of 0.999, and the relevant spectra are shown in Figure S18 (Supporting Information). It can be found that with the increase of the endotoxin concentration, the q‐BIC peaks show obvious red‐shifts for all experiments. The endotoxin concentration as low as 0.01 EU mL^‐1^ can be detected, which is much better than most commercial endotoxin detection kits (Figure 5g and Table S3, Supporting Information). Meanwhile, when the endotoxin concentration is larger than 1 EU mL^‐1^, the specific binding between endotoxin and aptamer will tend to be saturated, and the dielectric environment around the metasurface will tend to be stable, which is the reason for the slope reducing of the shift curve. Therefore, our studies demonstrate that the H‐OP architecture possesses a large second‐order surface sensitivity with a slow decay rate, showing a potential for identifying low‐concentrated small biological molecules.

## 3 Conclusion

A series of 2.5D architectures‐based plasmonic SP‐BIC metasurfaces have been constructed by using the BP‐AAO template technique. Simulation and experimental results confirm that the sole OP architectures could produce robust q‐BIC resonances even with a large AF. Moreover, with the assistance of the DNN, “BIC cubes” were generated by adjusting 5D structure parameters to quickly predicate the desired SP‐BIC metasurfaces. The H‐OP (Au‐SiO_2_) architecture with robust high *Q*‐factor and FoM is proposed and realized, which is very suitable for biosensing. For example, the detection limit of small molecules (e.g., endotoxin) can be as low as 0.01 EU mL^‐1^. Overall, our work reveals novel multi‐dimensional manipulation for new types of BIC metasurfaces that have the practical potential for many optical and optoelectronic device applications.

## 4 Experimental Section

### Definition of Polarizability

According to the electrostatics approximation,^[^ ^50^ ^]^ the polarizability (*p*) is defined by the geometry of plasmonic NP's semiaxes *a*, *b* and *c*, then the *p* could be written as follow:

(1) $$p_{i}=4\pi abc\frac{\varepsilon_{m}-\varepsilon_{d}}{3\varepsilon_{d}+3L_{i}\left(\varepsilon_{m}-\varepsilon_{d}\right)}$$

where *i* = 1, 2, or 3, meaning that the applied field is parallel to the *a*, *b* and *c* axes, respectively. *ε*_m_ and *ε*_d_ are the relative permittivities of metal and surrounding dielectric, and *L* is the shape factor. *L* can be calculated as follow:

(2) $$L_{i}=\frac{abc}{2}\int_{0}^{\infty }\frac{dq}{\left(x^{2}+q\right)f\left(q\right)}$$

where *i* = 1, 2 or 3, and the corresponding *x* = *a*, *b* or *c*, respectively, and *f*(*q*) = {(*a* ^2^ + *q*)(*b* ^2^ + *q*)(*c* ^2^ + *q*)}^1/2^. *q* is the amount of charge carried by a single NP.

### Fabrication of BP‐AAO Template

The fabrication process of the BP‐AAO template mainly includes the following steps: A Ni film with periodic nanopillars (*T* = 800 nm) was used to imprint the electropolished Al foils under the pressure of 15 kN cm^‐2^ for 3 min, then arrayed nanodents with a spacing of 800 nm was obtained on the surface of Al foil. A voltage of 320 V was used to anodize the imprinted Al foil in a mixed solution (4 g citric acid, 200 mL ethanediol, 200 mL H_2_O, and 10 mL 0.1 wt% H_3_PO_4_) at 30 °C for 2 h to achieve arrayed nanopores (A‐pores). 5 wt% H_3_PO_4_ solution was used to enlarge the A‐pores for 2 h, then a thin TiO_2_ layer was deposited into the A‐pores by employing the D100‐4882 Yaona Electronics ALD system for protecting the A‐pores from chemical etching. The ALD was carried out at 150 °C with 70 cycles consisting of 0.5 s C_12_H_28_O_4_Ti, 8 s N_2_ purge, 0.1 s H_2_O, and 8 s N_2_ purge, then the ion milling system (IM4000plus) was employed to mill off the top surface of BP‐AAO template to ensure that the B‐pores can be opened completely. PMMA solution was coated on the surface of BP‐AAO template. The unoxidized Al was removed in a mixed solution (1.5 wt% CuCl_2_ and 53.2 wt% HCl), then the PMMA layer was dissolved in acetone. Finally, the BP‐AAO template was immersed in 0.1 m NaOH solution for 30 min at room temperature, and a new set of pores (B‐pores) were obtained at the fourfold junction sites of A‐pores. At this point, the BP‐AAO template that is used in Figure S1 (Supporting Information) was prepared completely.

### Fabrication of the 2D IP and 2.5D OP Architectures

The fabrication process of IP and OP architecture is similar and the only difference is opening the A‐pores before or after the physical vapor deposition (PVD) process. When the barrier layer of the A‐pores was milled off before the PVD, A‐pores and B‐pores could be used for the deposition at the same time. So, the height of all NPs was the same, which can be used for the fabrication of IP architectures. When the barrier layer of A‐pores was milled off after the PVD, A‐pores could be used for one‐time deposition while B‐pores could be used for two times depositions, which can be used for the fabrication of OP architectures. Meanwhile, the size of A‐pores can be adjusted by the etching time in 5 wt% H_3_PO_4_ solution, while the size of B‐pores can be tailored by the etching time in 0.1 mol L^‐1^ NaOH solution, individually.

### Measurement of Transmission Spectra

All spectra were measured by a UV–vis–NIR spectrophotometer (JASCO V‐770). Non‐polarized light source with normal incidence was employed to illuminate all metasurfaces, and the size of the light spot used for measurement was about 40 mm^2^. The transmission spectra of IP and OP architectures were measured in the air. For the bulk sensitivity, surface sensitivity, and biosensing measurements, the H‐OP architecture was immersed in the glycerol solution, pure water, and PBS buffer solution subsequently by using the microfluidic chip.

### Simulation

Finite‐difference time‐domain (FDTD) simulation was carried out with the Lumerical FDTD software package, the mesh is set as 5 nm in our simulations after a convergence test. Periodic boundary conditions were applied to form the 800 nm square periodic array in *x* and *y* directions and perfectly matched layers (PMLs) were set along the *z*‐direction. A planewave source, propagating along +*z* direction, was used for illumination. The optical properties of Au were taken from Johnson and Christy, the refractive index of SiO_2_ and quartz substrate were set as *n* = 1.5 and the refractive index of ALD deposited Al_2_O_3_ was measured by ellipsometer (Figure S15, Supporting Information).

### Deep Neural Network Algorithm

The DNN algorithm was constructed using typical fully connected neural network (FCNN) with 5 structure parameters (*d*_a_ *d*_b_ *h*_a_ *h*_b_ *l*) as input and 7 layers hidden layers were set with neurons number: 20 40 80 160 320 640 800, and a nonlinear function LeakyRelu was chosen in each hidden layer to efficiently tackle data representation, according to the practical performance of the DNN. The loss function of mean square error (MSE) was applied. And the final output layer had 1000 spectral points from 1100 nm to 1400 nm with 0.3 nm high resolution. From the numerical simulations, 12705 samples were collected and used 10164 of them for training and the remaining 2541 for testing, the results were shown in the text but the training data can be less as tried in the Supporting Information. The model is constructed under the open‐source machine learning framework of TensorFlow.

### Calculation of the Sensitivity Factor, Decay Length, and Second‐Order Surface Sensitivity

The analysis of the surface sensitivity is based on the following theoretical equation:^[^ ^60^ ^]^

(3) $$\Delta \lambda =m\times \left(n_{\mathrm{ads}}-n_{\mathrm{med}}\right)\times \left(1-e^{-2t/l_{d}}\right)$$

The relation between the peak shift Δ*λ* and the sensitivity factor *m* is given by this equation, where the *n*_ads_ is the adsorbate index (here, the adsorbate is the Al_2_O_3_, and *n*_ads_ = 1.63, Figure S15, Supporting Information), the *n*_med_ is the refractive index of the medium (here, the medium is the water, and *n*_med_ = 1.33), *t* is the thickness of Al_2_O_3_ grown by ALD and *l*_d_ is the decay length. The experimental values of *m* and *l*_d_ are obtained by nonlinear fitting of the experimental data with Equation (3), and the simulation values are obtained by fitting the data obtained by FDTD. Moreover, based on the sensitivity factor and the decay length, the second‐order surface sensitivity *S* can be calculated as follow:

(4) $$S=\frac{2m}{l_{d}}\times e^{-2d/l_{d}}$$

By substituting experimental data and simulation data into Equation (4), the experimental and simulation function images between second‐order sensitivity *S* and distance from Au surface *d* can be drawn respectively.

### Endotoxin Sensing Experiments

Before the biosensing experiments, metasurface samples were treated with oxygen plasma (HARRICK) with high power for 5 min, then the microfluidic chip was fabricated by integrating the PMMA outer shell with the metasurface samples. All the modifications and sensing experiments were carried out by using this chip in room temperature, and the 1 × PBS was used as the buffer solution. First, 5 mg mL^‐1^ DTSP (Sigma‐Aldrich) in reagent plus dimethyl sulfoxide (DMSO, Aladdin, AR) was passed through the microfluidic chip and it was incubated for 2 h to finish the chemical linkage completely. Since thiol functional group of DTSP has selective reaction toward Au, the DTSP can make link between Au NPs and aptamer (or BSA). Second, the deionized (DI) water was passed through the channel to clean the sample surface, and then 20 × 10^‐6^ m PBS solution of aptamer (synthesized by Sangon Biotech with the following sequences: 5′‐HS‐(CH_2_)_6_‐CTTCTGCCCGCCTCCTTCCTAGCCGGATCGCGCTGGCCAGATGATATAAAGGGTCAGCCCCCCAGGAGACGAGATAGGCGGACACT‐MB‐3′) was filled the channel and it was maintained for 3 h, after that, the PBS buffer was passed through to clean the sample surface. Third, 1 wt% BSA in PBS solution was filled the channel and it was maintained for 1 h, after that, using PBS buffer to clean the sample surface too. Finally, PBS solutions of endotoxin (Xiamen Bioendo Technology) with different concentrations (0.01, 0.1, 1, 5, and 10 EU mL^‐1^) were filled the channel and maintained for 30 min, after that, using PBS buffer to clean the sample surface. All transmission spectra were measured after the surface cleaning steps, and the spectra were recorded from three samples to ensure the reliability.

## Conflict of Interest

The authors declare no conflict of interest.

## Supporting information

Supporting Information

## Data Availability

The data that support the findings of this study are available from the corresponding author upon reasonable request.
