# Supplementary material for: Customizing 2.5D Out‐of‐Plane Architectures for Robust Plasmonic Bound‐States‐in‐the‐Continuum Metasurfaces
Source: Adv Sci (Weinh). 2023 Jan 3;10(7):2206236. doi: 10.1002/advs.202206236 (PMC9982570; doi:10.1002/advs.202206236)
Supplement: Supplementary file 1 — Supporting Information [file ADVS-10-2206236-s001.pdf]

ADVANCED SCIENCE
Open Access

Supporting Information

for *Adv. Sci.*, DOI 10.1002/advs.202206236

Customizing 2.5D Out-of-Plane Architectures for Robust Plasmonic Bound-States-in-the-Continuum Metasurfaces

Zichen Wang, Jiacheng Sun, Jiye Li, Lang Wang, Zishun Li, Xiaorui Zheng and Liaoyong Wen**

Supporting Information

Title:

Customizing 2.5D Out-of-plane Architectures for Robust Plasmonic Bound-States-in-the-Continuum Metasurfaces

Zichen Wang[1,2,3†], *Jiacheng Sun*[2,3†], *Jiye Li*[2,3], *Lang Wang*[2,3], *Zishun Li*[2,3], *Xiaorui Zheng*[2,3*] *and Liaoyong Wen*[2,3*]

[1]College of Information Science and Electronic Engineering, Zhejiang University, Hangzhou 310027, People's Republic of China.
[2]Research Center for Industries of the Future (RCIF), School of Engineering, Westlake University, Hangzhou 310030, Zhejiang, People's Republic of China.
[3]Key Laboratory of 3D Micro/Nano Fabrication and Characterization of Zhejiang Province, School of Engineering, Westlake University, Hangzhou 310024, People's Republic of China.

*Corresponding authors.
Email: xiaoruizheng@westlake.edu.cn (X.Z.); wenliaoyong@westlake.edu.cn (L.W.)
†These authors contributed equally to this work.

This PDF file includes:

Figures S1 to S18
Tables S1 to S3

Supplementary Figures

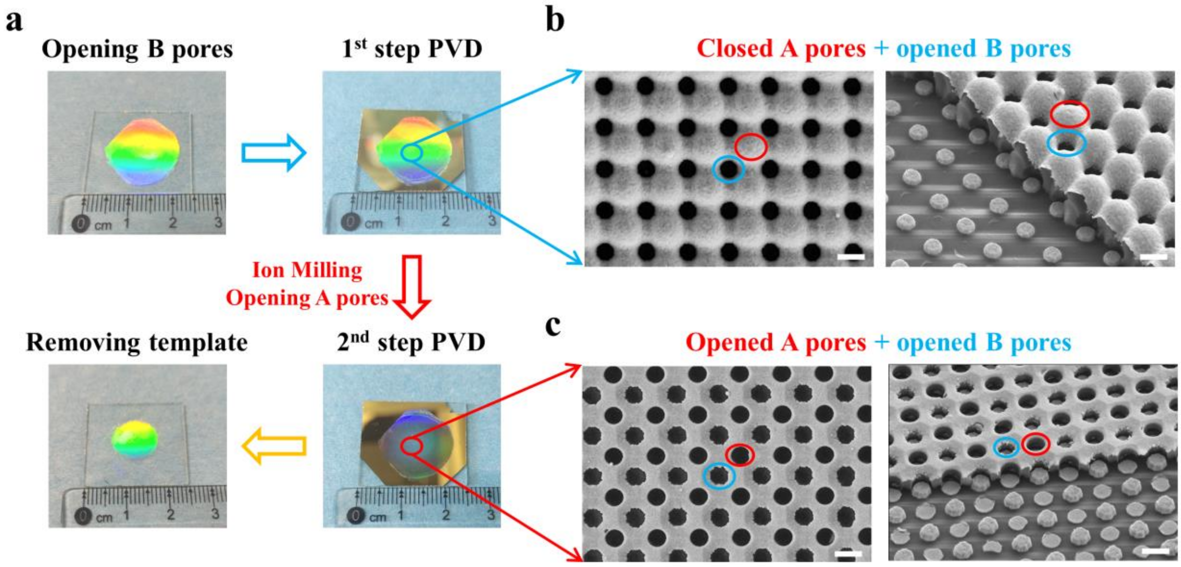

Figure S1. Fabrication of sole OP architecture. All scale bars: 300 nm. (a) Photographs of a representative sample after each step of treatment. (b) Top-down and tilted SEM images of the BP-AAO template with closed A-pores and opened B-pores, and the Au NPs arrays are tetragonal arranged with a period of 800 nm after 1st step PVD. (c) Top-down and tilted SEM images of the BP-AAO template with opened A- and B-pores, where the A-pores were opened by using ion milling, and binary tetragonal nested Au NPs arrays with OP architectures are successfully fabricated after 2nd step PVD.

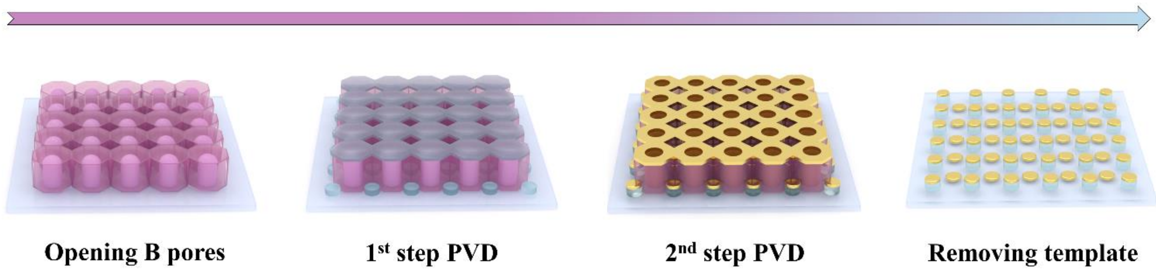

Figure S2. Schematic of fabrication process of H-OP architecture. The H-OP architecture with Δh and hybrid NPs-b can be fabricated by using two different evaporator sources during the twice PVD processes.

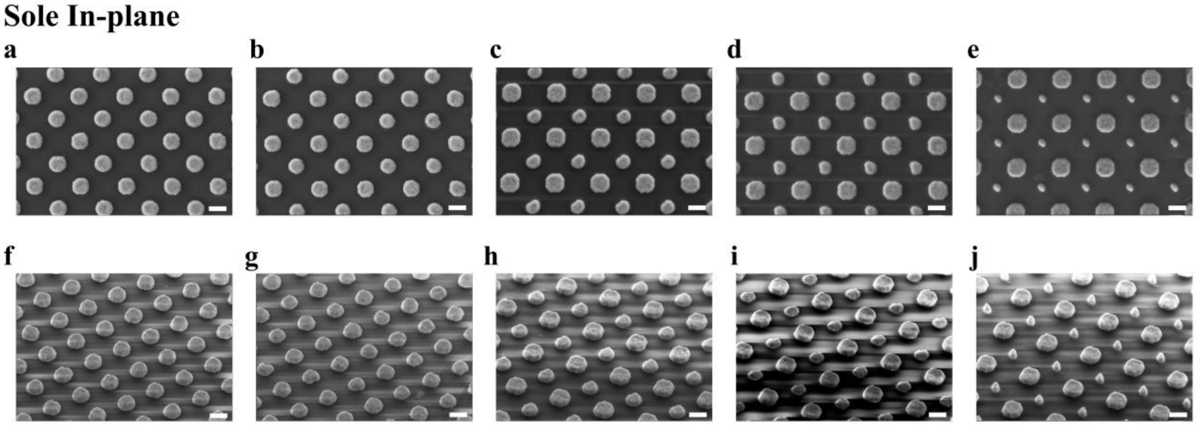

Figure S3. SEM images of sole IP architectures. (a-e) Top-down view of sole IP architectures with different IP AF, where the diameters of NPs-a and NPs-b are about 300/300 nm, 280/320 nm, 250/340 nm, 210/350 nm and 130/350 nm, respectively. (f-j) Tilted angle view of sole IP architectures with the same OP AF, where the heights of NPs-a and NPs-b are both about 160 nm for all samples. scale bars: 300 nm.

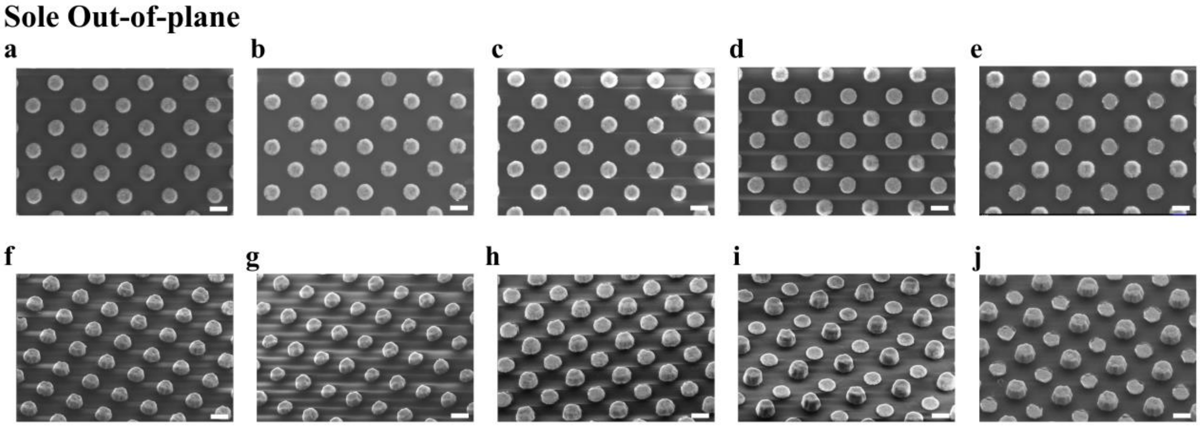

Figure S4. SEM images of sole OP architectures. (a-e) Top-down view of sole OP architectures with same IP AF, where the diameters of NPs-a and NPs-b are both about 300 nm for all samples. (f-j) Tilted angle view of sole OP asymmetric metasurfaces with different OP AF, where the heights of NPs-a and NPs-b are about 160/160 nm, 120/160 nm, 80/160 nm, 40/160 nm and 20/160 nm, respectively. scale bars: 300 nm.

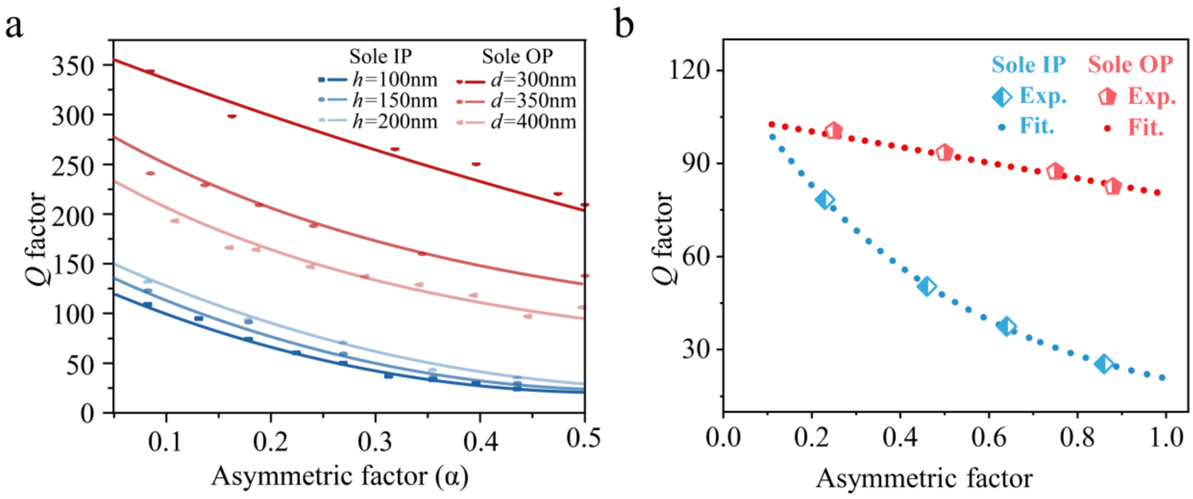

Figure S5. (a) Calculated and (b) experimental variation trends of *Q*-factors with the increasing of $AF_{(V)}$ for sole IP and OP architectures. The $AF_{(V)}$ is the change of volume difference, namely $(V_b-V_a)/V_b$, where the volume *V* of the pillar can be estimated by $h\pi(d/2)$^2 in the IP and OP metasurfaces. Therefore, for the sole IP metasurface, due to the heights of pillars are the same, $AF_{(V)}$ can be simply expressed as Δd^2/d_b^2 (Δd^2 = d_b^2 - d_a^2); while, for the sole OP metasurface, $AF_{(V)}$ can still be expressed as $\Delta h/h_b$ because the pillars have the same diameter.

For the blue lines in **Figure S5(a)**: sole IP architectures with different *h* from 100 nm to 200 nm fitted by exponential curves: Q = 195 exp($-\alpha$/0.172) + 8.33 (h = 100 nm); Q = 189 exp($-\alpha$/0.239) – 0.18 (h = 150 nm); Q = 201 exp($-\alpha$/0.22) + 6.52 (h = 200 nm). The red lines in **Figure S5(a)**: sole OP architectures with different *d* from 300 nm to 400 nm fitted by exponential curves: Q = 524.56 exp($-\alpha$/1.39) – 149.94 (d = 300 nm); Q = 216.36 exp($-\alpha$/0.37) + 79.41 (d = 350 nm); Q = 193.86 exp($-\alpha$/0.33) + 57.23 (d = 400 nm). It can be seen that the simulated *Q*-factor of the sole IP metasurface still decreases exponentially with the increase of AF (**Figure S5a**). Most importantly, the overall trends of *Q*-factor change of sole IP and OP metasurfaces are consistent with our previous results. The sole OP metasurfaces still possess better *Q*-factor robustness than the sole IP metasurfaces, and this conclusion can still be supported by our experimental results (**Figure S5b**).

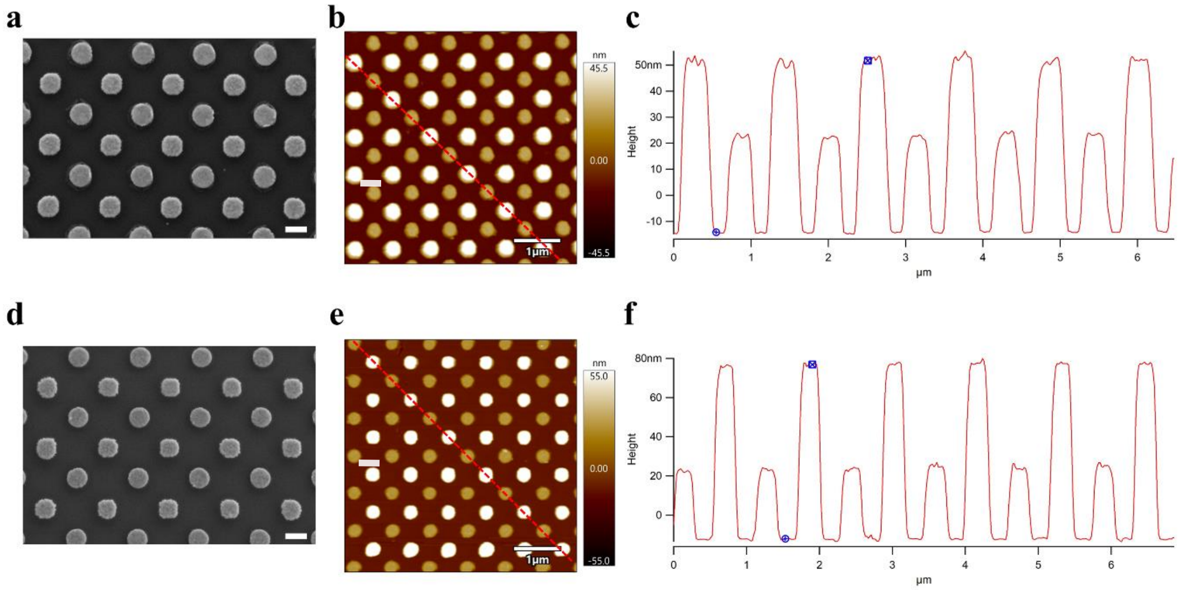

Figure S6. SEM and AFM images of H-OP architectures. (a, d) Top-down view of the H-OP architecture with different H-OP AF, where the diameters of NPs-a and NPs-b are both about 300/300 nm. (b, e) 2D AFM images and (c, f) the height profiles scanned across the profile lines (red lines) for different positions of the H-OP architecture. It can be seen that for these two H-OP architectures, the heights of NPs-a and NPs-b are about 40/65 nm and 40/90 nm, respectively. So, their H-OP AF are 0.38 and 0.56, respectively. All scale bars for SEM: 300 nm.

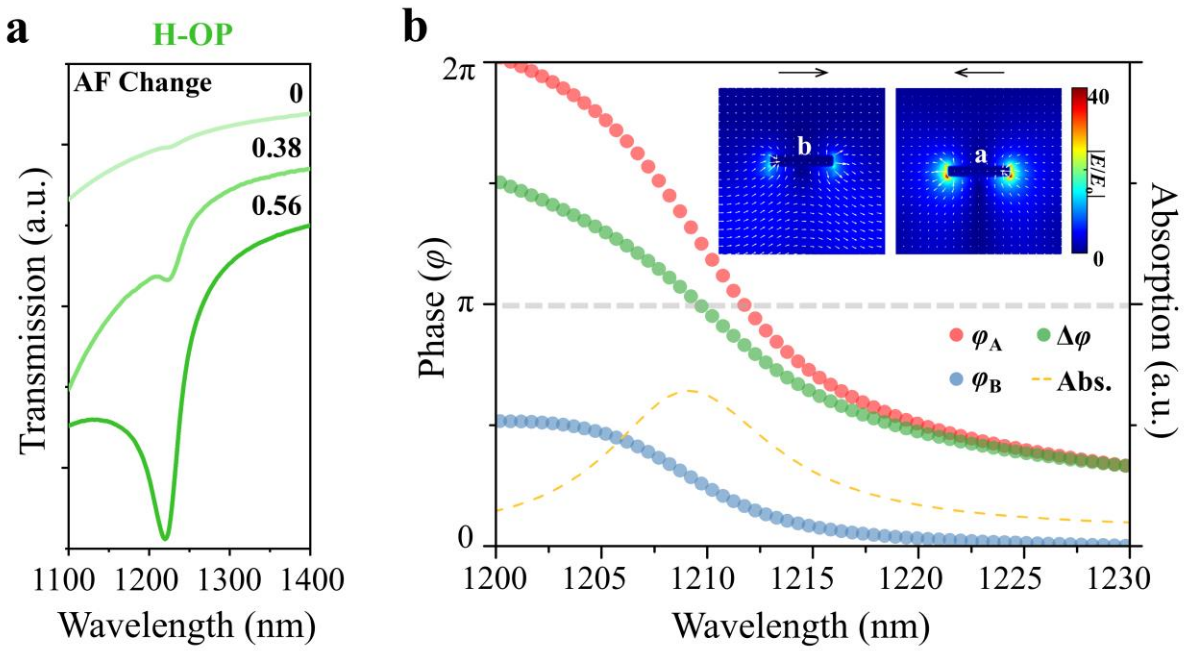

Figure S7. Optical characteristics of H-OP architectures. (a) Experimental transmission spectra of H-OP architectures with different AF (corresponding α are 0, 0.38, and 0.56, respectively). (b) Calculated phase variations of the NPs-a and NPs-b arrays under irradiation from the substrate. The correlative transmission spectra indicate that with α increases, the H-OP architectures represent a similar evolution law to IP and OP architectures, where the FWHMs of transmission peaks widen gradually, presenting typical SP-BIC characteristics. To reveal the inherent mechanism of the q-BIC excitation in such H-OP architectures, we calculated the phase variation of the NPs-a and NPs-b under irradiation from the substrate and the results show a classic destructive interference feature between the two sets of NPs (Fig. S6 (b)). The lifted NP-b with a relatively weak electric field enhancement reveals a gradual phase change around the resonance within π, as a discrete mode; while the grounded NP-a with a much stronger electric field enhancement obtains a jumpy phase change around the resonance beyond π, as a continuous mode. As a result, the accumulated phase differences between the two sets of NPs reach π at the resonate wavelength (~1200 nm) and exhibit opposite electric vectors, forming the q-BIC resonance.

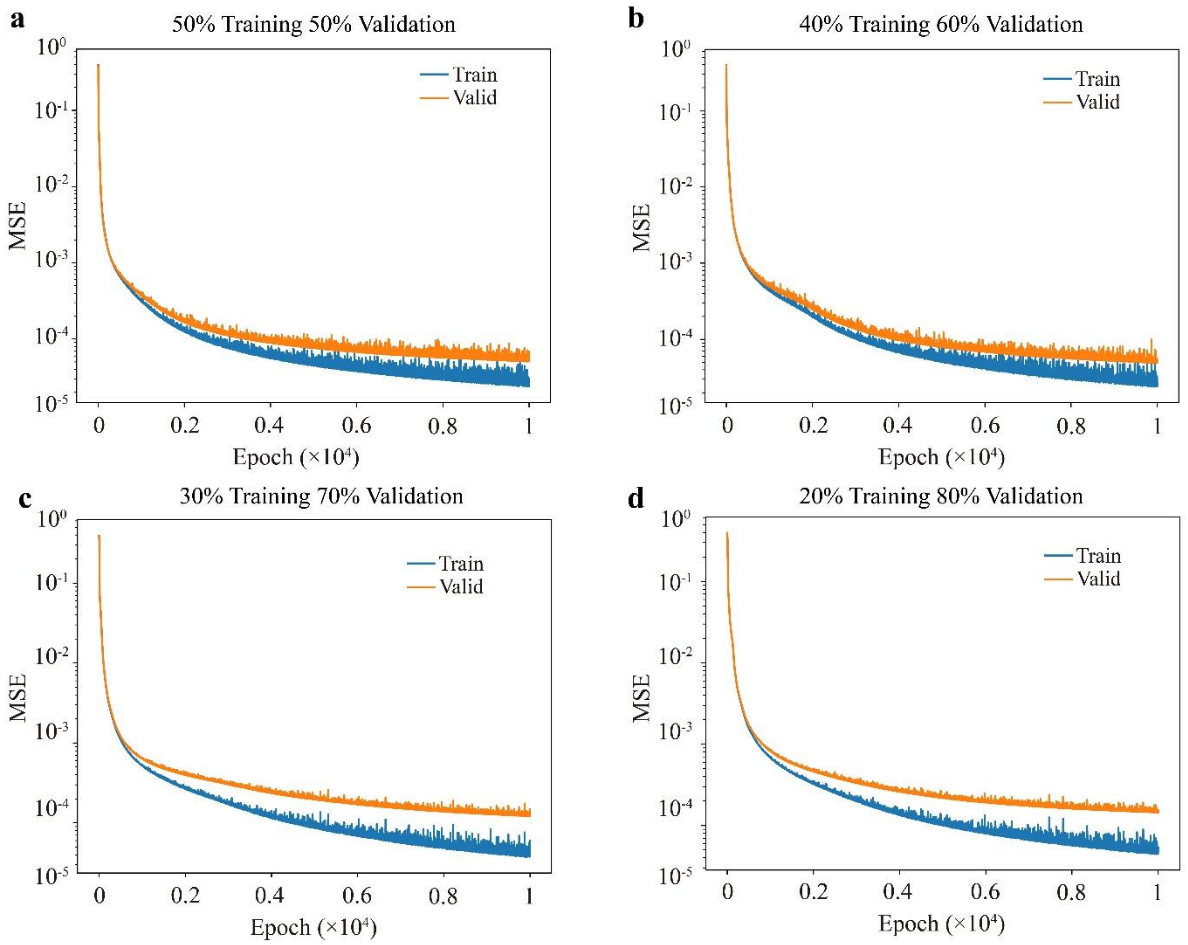

Figure S8. Training and validation curves of the different training data. (a-d) The total data set has 12705 simulation results, indicating that a smaller data set for the training: 50%, 40%, 30%, and even 20%, are also sufficient to obtain fairish learning results with the DNN.

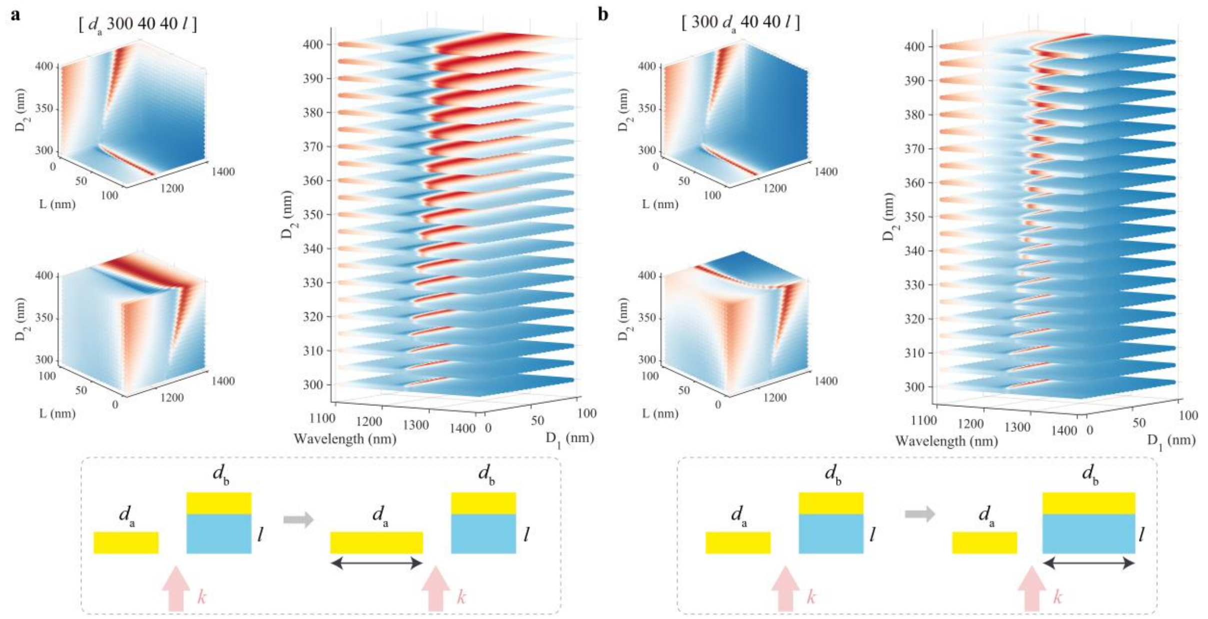

Figure S9. Multi-dimensional manipulation of H-OP architectures. (a) Predicted BIC cubes of H-OP architectures with the structural parameters: [d_a 300 40 40 l]. It is obvious that with the increase of d_a, the quasi-BIC resonance will become broaden dramatically. (b) Predicted BIC cubes of H-OP architectures with the structural parameters: [300 d_b 40 40 l]. The increase of d_b efficiently surpasses the broadening of quasi-BIC resonance, keeping the Q-factor very robust.

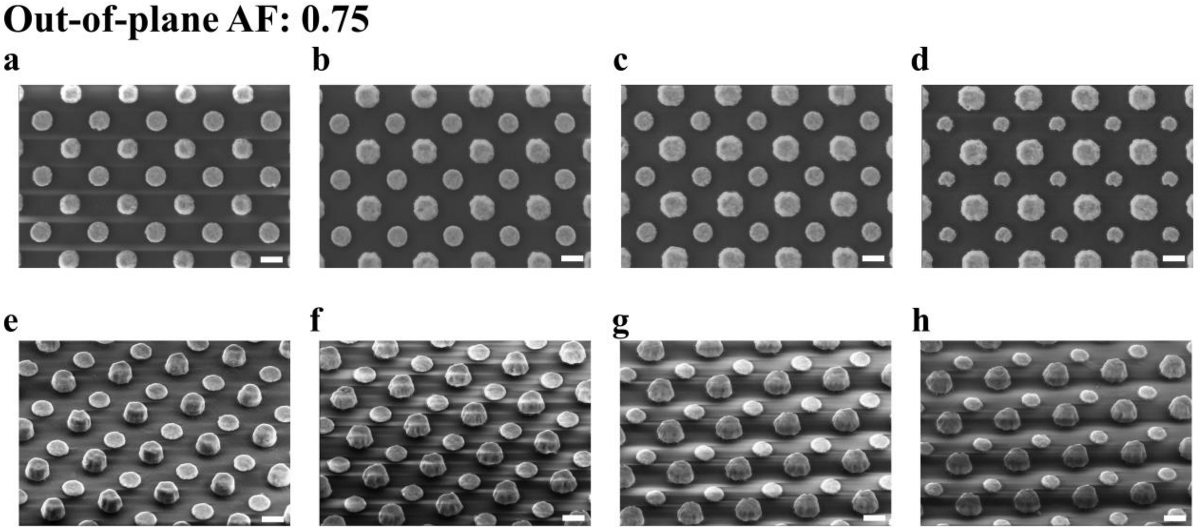

Figure S10. SEM images of OP architectures (OP AF = 0.75). (a-d) Top-down view of OP architectures with different IP AF, where the diameters of NPs-a and NPs-b are about 300/300 nm, 285/330 nm, 265/340 nm, and 230/350 nm, respectively. (e-h) Tilted angle view of OP architectures with a same OP AF of 0.75, where the heights of NPs-a and NPs-b are about 40/160 nm for all samples. scale bars: 300 nm.

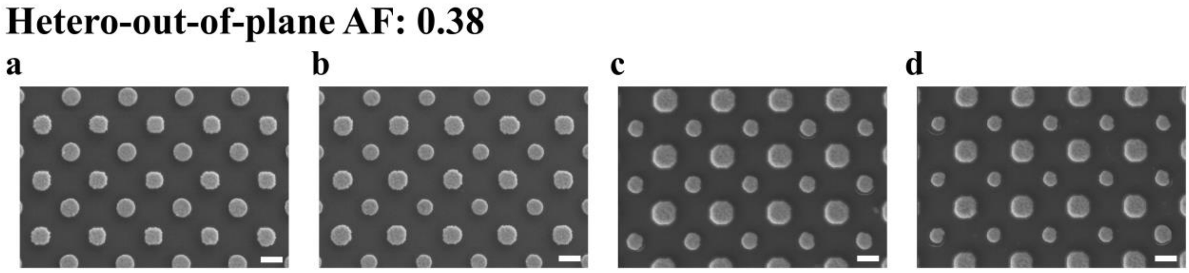

Figure S11. SEM images of H-OP architectures with a H-OP AF of 0.38. (a-d) Top-down view of H-OP architectures with different IP AF, where the diameters of NPs-a and NPs-b are about 300/300 nm, 280/320 nm, 250/340 nm, and 210/350 nm, respectively. the heights of NPs-a and NPs-b are both about 40/65 nm for all samples. Scale bars: 300 nm.

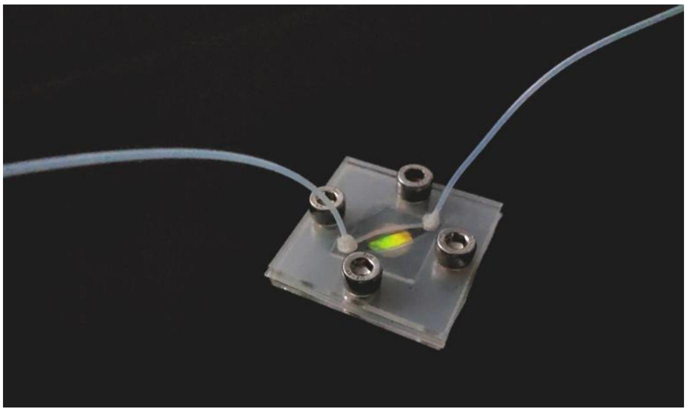

Figure S12. Photograph of the PMMA microfluidic chip used in our experiments.

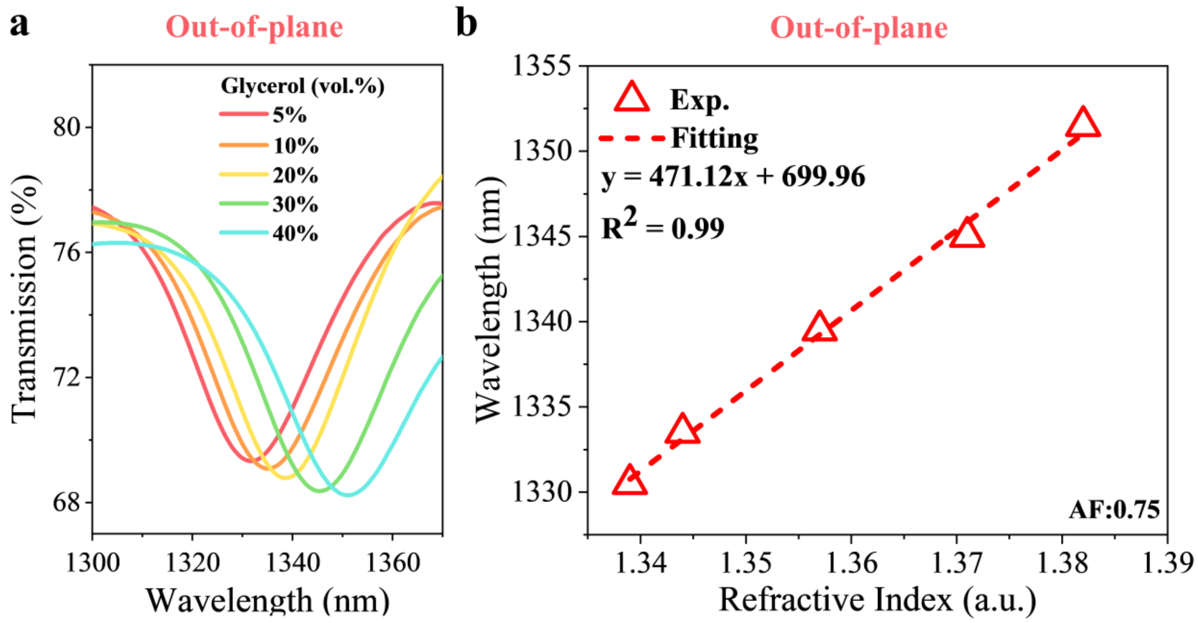

Figure S13. Sole OP architecture (OP AF = 0.75) for bulk refractive index sensing (glycerol solution). (a) Peak shifts of q-BIC resonance in different concentrations of glycerol solution (5-40% vol.%). (b) q-BIC resonance wavelength shifts as a function of RI variation. Dotted lines indicate the linearity response.

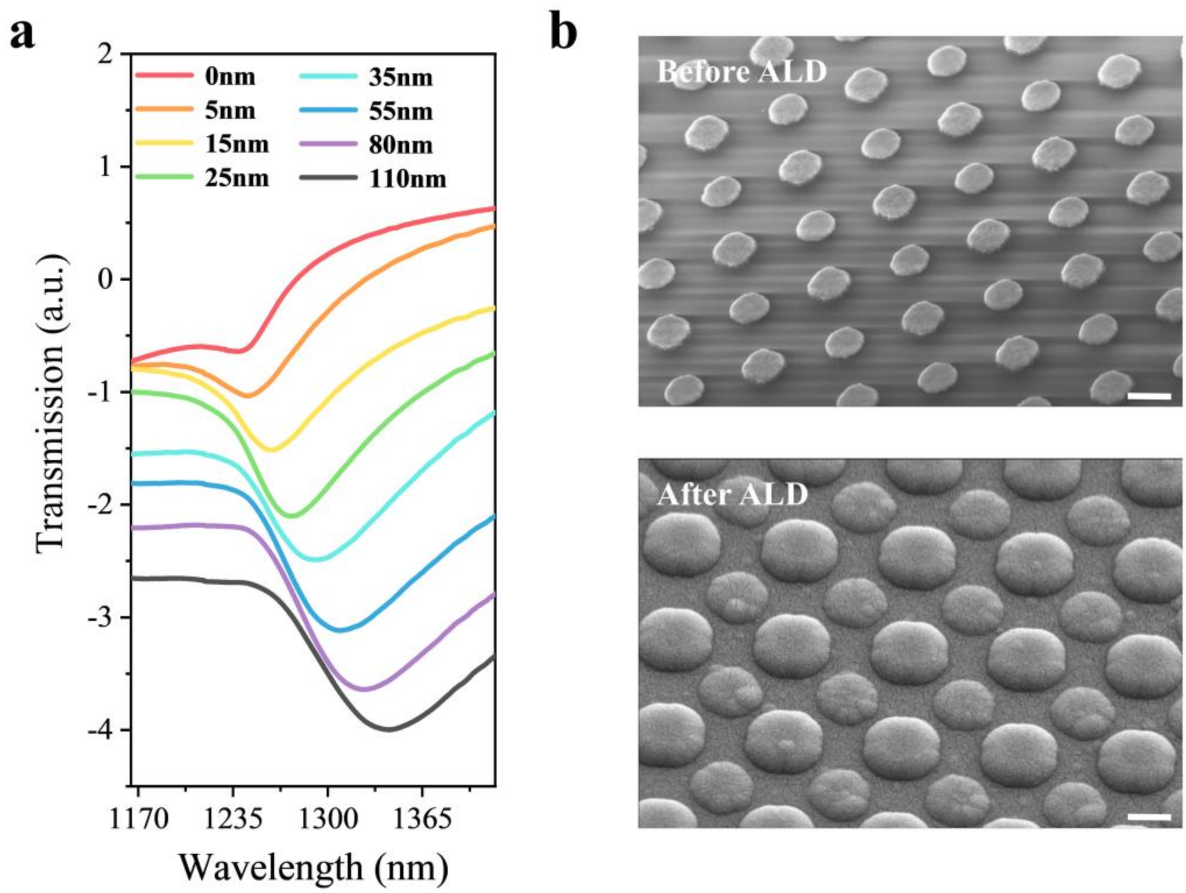

Figure S14. Transmission spectra (a) and SEM images (b) of H-OP architectures (H-OP AF = 0.38) before and after covered with ALD Al_2O_3. All scale bars: 300 nm.

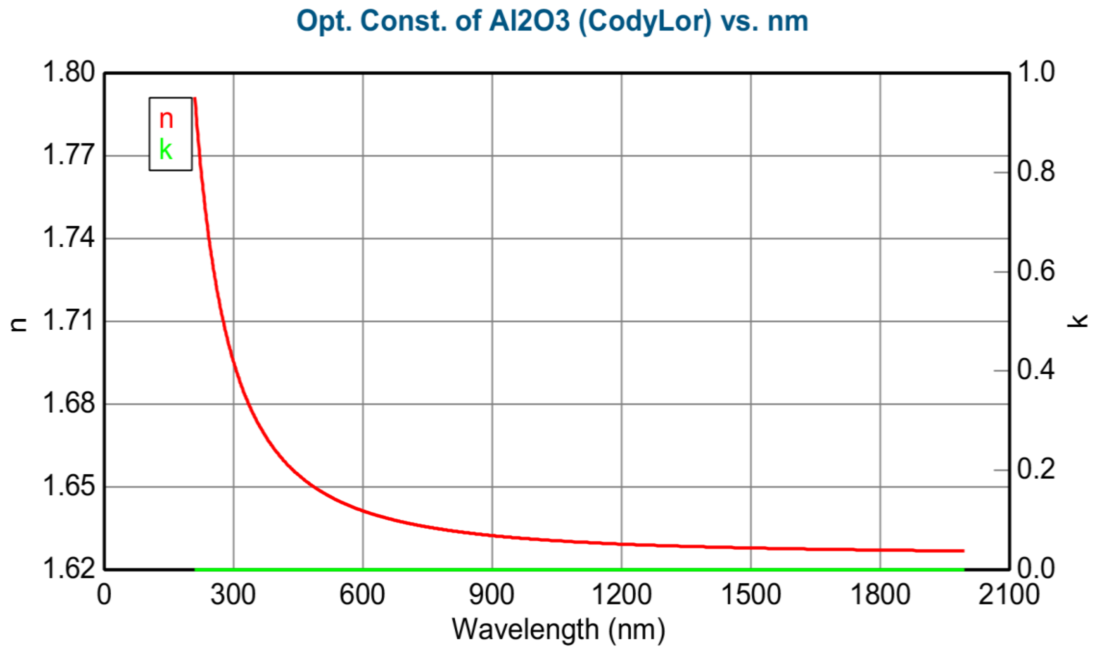

Figure S15. The optical constant of Al_2O_3 layer measured by the ellipsometer (RC2 XI+). The refractive index (*n*) of Al_2O_3 layer at the wavelength near 1300 nm is about 1.63, and the extinction coefficient (*k*) is about 0 in the range of measured wavelengths.

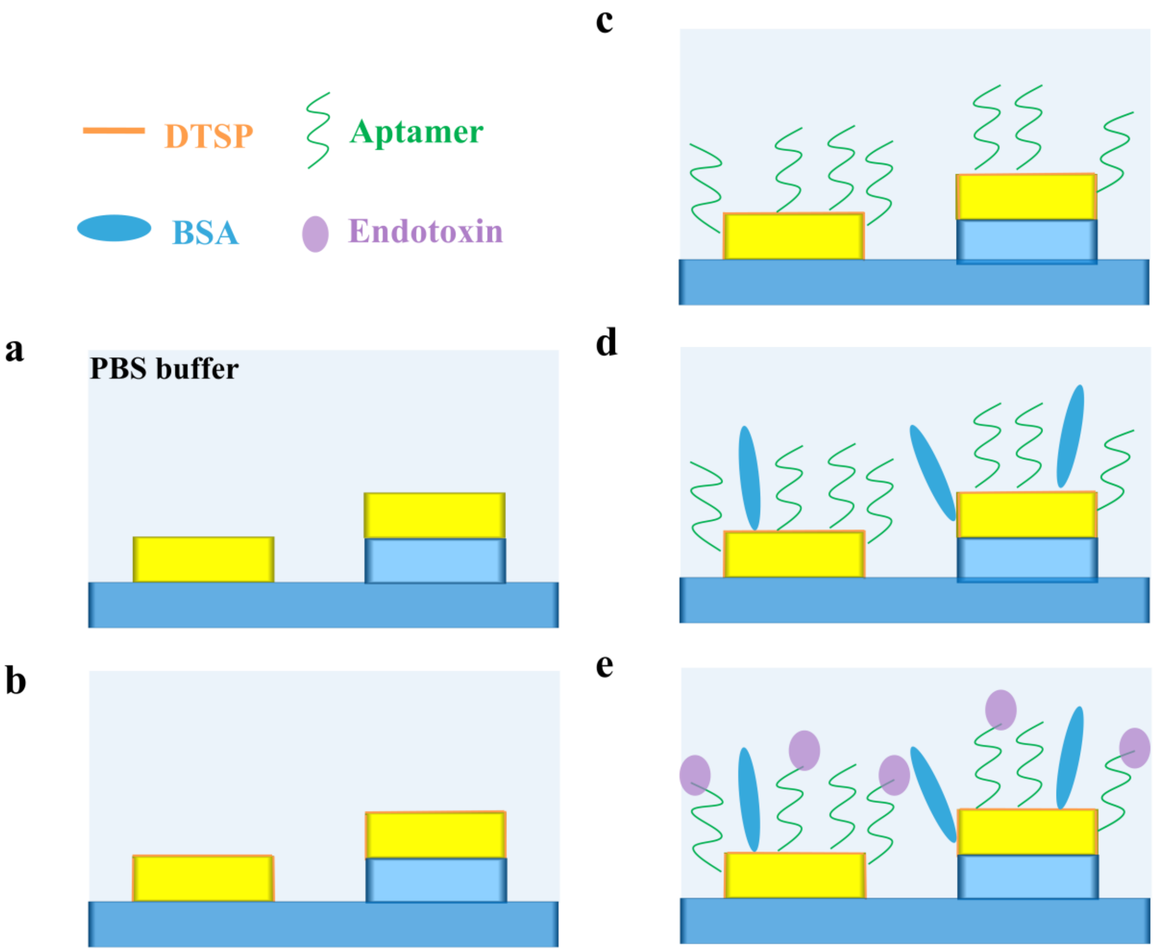

Figure S16. Schematic diagrams of chemical modification and endotoxin specific binding. (a) 1 × PBS was used as the buffer solution. (b) DTSP was coated on the Au surface. (c) The aptamer with specific base sequences was combined with the DTSP on the Au surface. (d) BSA was filled on the Au surface to avoid the subsequent nonspecific binding. (e) Specific binding between the endotoxin and the aptamer was achieved.

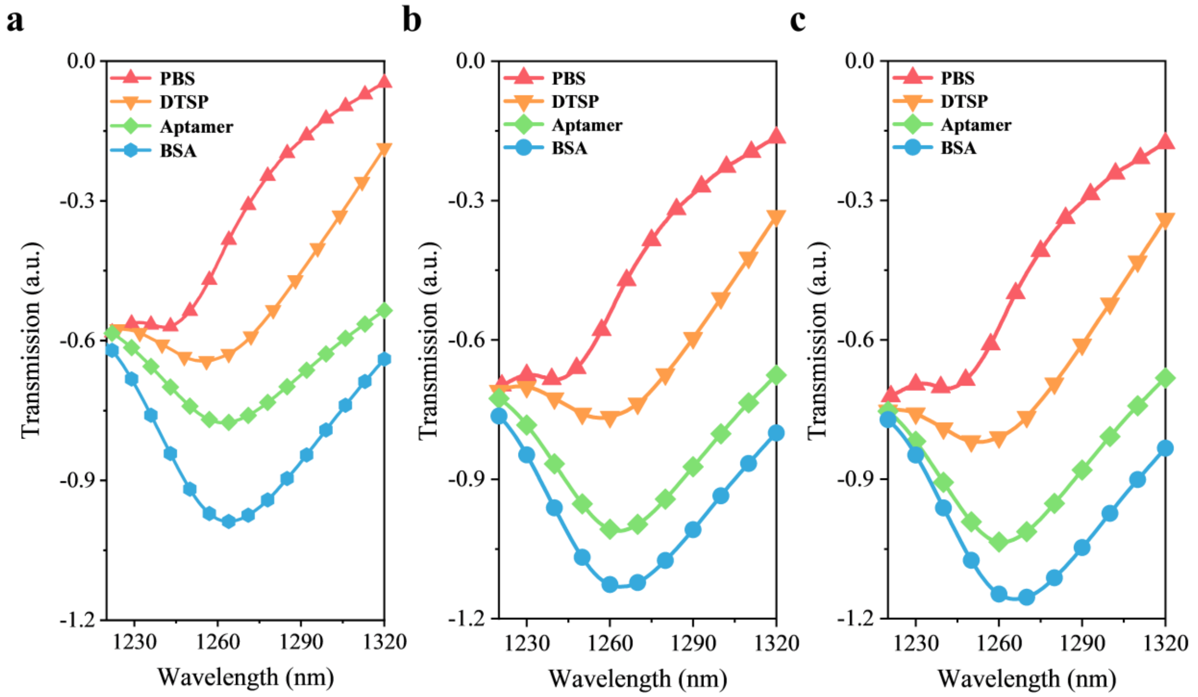

Figure S17. Transmission spectra of H-OP architectures (H-OP AF: 0.38) after different steps of modification (three groups of experiments).

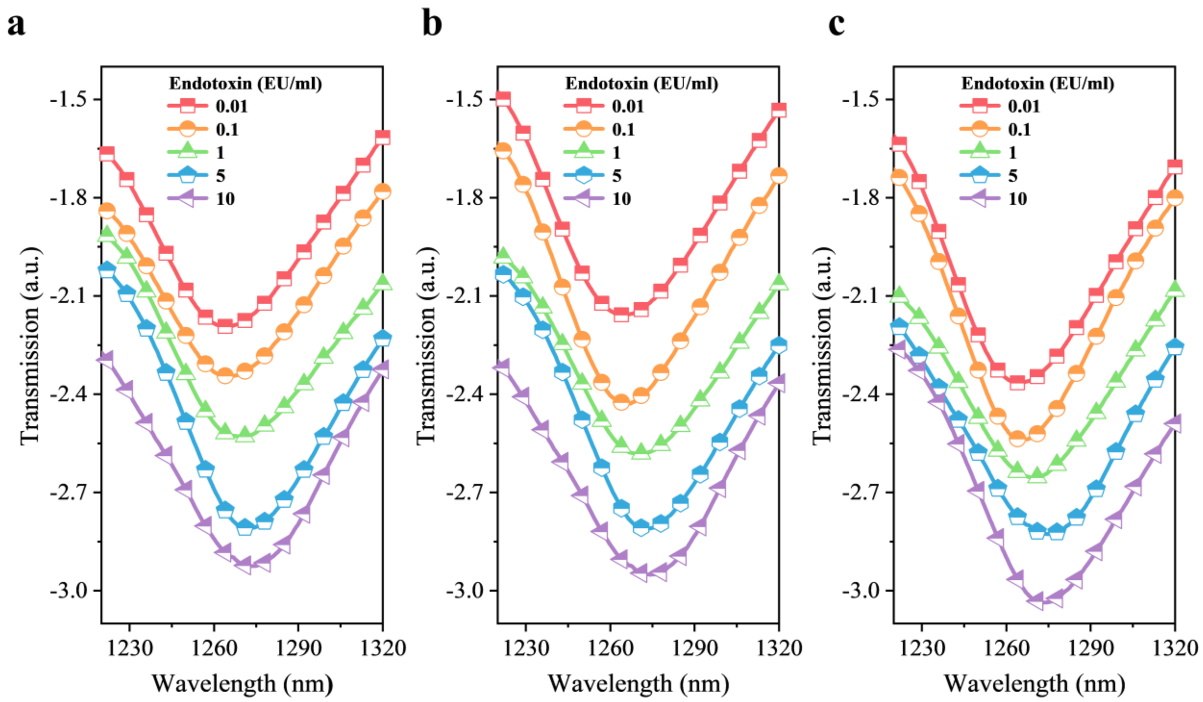

Figure S18. Transmission spectra of the modified H-OP architectures (H-OP AF: 0.38) in different concentrations of endotoxin solution (three groups of experiments).

Supplementary Tables

Table S1. The mean values and errors of associated resonance shifts in three groups of experiments about the surface modification (unit: nm).

Mod. / No.	PBS	DTSP	Aptamer	BSA
1	18.3	32.9	38.9	40.9
2	17	33.9	39.9	40.4
3	19.3	31.9	37.9	41.4
Av.	18.2	32.9	38.9	40.9

Table S2. The mean values and errors of associated resonance shifts in three groups of experiments about the binding of analytes (unit: nm).

Conc. (EU/ml) / No.	0.01	0.1	1	5	10
1	0.6	1.2	4.3	9.1	9.5
2	0.5	1.4	5.8	9.6	10.2
3	1.1	2	5.2	10.2	10.5
Av.	0.7	1.5	5.1	9.6	10.1

Table S3. comparison about the representative commercialized kits and our H-OP metasurface sensors.

Company	Kit Name	Method	Detection Time	Sensitivity (EU/ml)
Charles River Laboratories Inc.[1]	Endosafe® 50-test vial (5.2ml)-R11025	sol-gel	~1 h	0.25
	Endosafe® 10-test vial (1.2mL)-R11012			0.125
	Endosafe® Gel-Clot LAL Single-Test Vial (0.2 mL)-R13006			0.06
	KTA-50-test Vial (5.2 mL)-R15015	photometry		0.015
	Endosafe® Card-PTS2005F/PTS20005F	colorimetry	15 min	0.05/0.005
Youzre Biotech Co., LTD[2]	EndoLISA®	photometry	> 90 min	0.05
	EndoZyme®		——	0.005
Gen Script Biotech Co., LTD[3]	ToxinSensor™ Single Tests Kit with Standard-L00857-40	sol-gel	~1 h	0.06
	ToxinSensor™ Single Tests Kit with Standard-L00858-40	photometry	——	0.125
Zhanjiang A&C Biological Co., LTD[4]	RT065030/RT065125	sol-gel	——	0.03/0.125
	KT125030	photometry		0.03
Amyjet Scientific Co., LTD[5]	EndoAlert Endotoxin Plate Kit - KMA-0100	photometry	~1 h	0.01
Associates Of Cape Cod Co., LTD[6]	Pyrosate® KIT-PSD030/PSD250	sol-gel	~30 min	0.03/0.125
**Our H-OP metasurface sensors**		**BIC resonance**	**30 min**	**0.01**

Website informations：

[1] https://www.criver-microbial.cn/

[2] http://www.esepara.com/index-cn.html

[3] https://www.genscript.com.cn/

[4] http://www.zacb.com/zacb/product/endotoxin-detection/

[5] https://www.amyjet.com/featured/Rockland-LPS.shtml

[6] https://www.chem17.com/st100484/product_35800616.html
